# Phase Angle Is a Marker of Muscle Quantity and Strength in Overweight/Obese Former Athletes

**DOI:** 10.3390/ijerph18126649

**Published:** 2021-06-21

**Authors:** Catarina N. Matias, Francesco Campa, Catarina L. Nunes, Rubén Francisco, Filipe Jesus, Miguel Cardoso, Maria J. Valamatos, Pedro Mil Homens, Luís B. Sardinha, Paulo Martins, Cláudia Minderico, Analiza M. Silva

**Affiliations:** 1 Bioperformance & Nutrition Research Unit, Bettery S.A., 2740-262 Lisbon, Portugal; katarina_matias@hotmail.com; 2 CIDEFES-Universidade Lusófona, 1749-024 Lisboa, Portugal; 3 Exercise and Health Laboratory, CIPER, Faculdade Motricidade Humana, Universidade Lisboa, Estrada da Costa, 1499-688 Cruz-Quebrada, Portugal; catnunes94@gmail.com (C.L.N.); ruben92francisco@gmail.com (R.F.); fastj96@gmail.com (F.J.); m73cardoso@gmail.com (M.C.); lbsardinha55@gmail.com (L.B.S.); cminderico@gmail.com (C.M.); analiza.monica@gmail.com (A.M.S.); 4 Department for Life Quality Studies, Università degli Studi di Bologna, 40126 Rimini, Italy; 5 Neuromuscular Research Lab, CIPER, Faculdade Motricidade Humana, Universidade Lisboa, Estrada da Costa, 1499-688 Cruz-Quebrada, Portugal; mjvalamatos@fmh.ulisboa.pt (M.J.V.); pmilhomens@fmh.ulisboa.pt (P.M.H.); 6 Laboratory of Sport Psychology, Faculdade de Motricidade Humana da Universidade de Lisboa, 1499-002 Cruz-Quebrada, Portugal; pmartins@fmh.ulisboa.pt

**Keywords:** body composition, bioelectrical impedance, skeletal muscle, strength, phase angle

## Abstract

Background: An increasing body of evidence indicates that the phase angle (PhA) can be applied as a marker of nutritional status, disease prognosis, and mortality probability. Still, it is not known whether PhA can be used as an indicator of muscular quantity and strength and maximal aerobic capacity in overweight/obese former highly active individuals, an understudied population. This study aimed to analyze the association between PhA with skeletal muscle mass, maximal isometric strength, and maximal aerobic capacity through VO2max, in overweight/obese and inactive former athletes. Methods: Cross-sectional information of 94 (62 males) former adult athletes (age: 43.1 ± 9.4 years old; body mass index: 31.4 ± 4.8 kg/m^2^) taking part in a weight-loss clinical trial was analyzed. Total fat and fat-free mass were determined by dual-energy X-ray absorptiometry, while skeletal muscle mass was predicted from appendicular lean soft tissue. Values for upper- and lower-body maximal isometric strength were assessed by handgrip and leg press dynamometry. VO2max was determined by indirect calorimetry through a graded exercise test performed on a treadmill. Results: PhA was associated with skeletal muscle mass (r = 0.564, *p* < 0.001), upper-body strength (r = 0.556, *p* < 0.001), lower-body strength (r = 0.422, *p* < 0.001), and VO2max (r = 0.328, *p* = 0.013). These relationships remained significant for skeletal muscle mass (β = 2.158, *p* = 0.001), maximal isometric strength (upper-body: β = 2.846, *p* = 0.012; low-er-body: β = 24.209, *p* = 0.041) after adjusting for age, sex, and fat mass but not for VO2max (β = −0.163, *p* = 0.098). Conclusion: Our findings indicated that former athletes with higher values of PhA exhibited greater muscle mass and strength, despite sex, age, and body composition, which suggests that this simple raw BI parameter can be utilized as an indicator of muscle quantity and functionality in overweight/obese former athletes.

## 1. Introduction

When the Irish writer George Bernard Shaw wrote “We don’t stop playing because we grow old, we grow old because we stop playing,” he probably was not referring to former athletes, but it may have inspired many of them to continue the practice of physical activity after the end of their career. It is well known that the practice of regular physical activity has a beneficial effect on muscle quality and strength, as well as on cardiorespiratory fitness, improving the quality of life and preventing the onset of several pathologies [1]. On the contrary, the neglect of physical activity and a sedentary life leads to a worsening of health status and poor quality of life [2].

It has been reported that an alarming number of former athletes adopt a sedentary lifestyle after their retirement from competition, resulting in a remodeling of body composition and a decline in physical performance [3,4]. Body composition parameters are related to the health status and physical performance in athletes, as well as in the overall population [5,6]. Among these, the quality and quantity of the muscle mass are informative parameters. In fact, a reduction in muscle mass can compromise muscle strength and physical function, and the reduction in both muscle mass and strength is considered a key factor in the diagnosis of sarcopenia [2]. In addition, fitness-related aspects including maximal aerobic power (VO2max) are related to the quality of life and are included among the characteristics that worsen with both aging and sedentary life [7,8].

The reference methods used in laboratory routine analysis to assess body composition, strength, and cardiorespiratory capacity require expensive instruments and qualified personnel. Among these methods, dual-energy X-ray absorptiometry (DXA) is used to evaluate the appendicular lean soft tissue mass and skeletal muscle mass [9], while the strength assessment requires the use of dynamometers or specific machines. Additionally, VO2max involves the measurement of oxygen uptake (VO2) using expired gas indirect calorimetry during incremental exercise to volitional exhaustion [10].

Scientific research has always been focused on identifying biomarkers that can also be used in the field context. The bioelectric phase angle (PhA) has been suggested as an indicator of nutritional status, cellular integrity, and muscle quality [2,6,11,12,13,14]. PhA has recently been identified as a predictor of the competitive level and related to sports performance in athletes [15,16]. PhA can quickly and easily be calculated by low-cost bioelectrical impedance analyzers, as the arctangent of bioelectric reactance and resistance [6,17]. It has been suggested that, since the increase in PhA can be explained by an increase in muscle cell volume, it might be more sensitive in detection muscle size and function than lean soft tissue estimates [6]. Nevertheless, it is not yet known whether PhA can be used as an indicator of muscle quantity and strength, and maximal aerobic power in overweight/obese former athletes, who also represent a poorly studied population. Skeletal muscle mass and strength, as well as the aerobic fitness level, are relevant physical fitness parameters underlying sports performance [6]. In fact, skeletal muscle mass represents functional mass and positively contributes to strength production [18], while an optimal level of aerobic fitness contributes to generate and maintain power output during repeated high-intensity efforts and to recover [19]. However, at the end of the athletic career, these parameters undergo a decline, a process that may also be accelerated if body fat becomes high, as observed in former elite athletes [4,20,21]. In addition, many athletes who retired from their sports careers do not sustain a regular exercise routine, contributing to a decrement in physical fitness [22,23]. An undesired weight gain among former athletes might be expected given the new lower energy requirements. The resulting weight gain, particularly by increasing fat mass, increases the risk of emerging obesity-related adverse health effects. Indeed, fat mass is considered as a nonfunctional mass, with increasing amounts hidden mechanically and metabolically by sports performance and affecting negatively thermoregulation, physical functioning, and general health [23,24,25].

Therefore, the purpose of this investigation was to evaluate whether PhA can be identified as a predictor of (i) skeletal muscle mass derived from DXA, (ii) isometric upper and lower strength measured with dynamometers, and (iii) VO2max evaluated with indirect calorimetry. Based on the known relationships between PhA and body composition, we hypothesized that PhA could be suggested as a predictor for muscle quantity and strength, as well as cardiorespiratory capacity, in former athletes.

## 2. Methods

### 2.1. Participants and Study Design

The present study used cross-sectional data collected during the baseline of the project Champ4Life (NCT03031951), a clinical trial that aims to analyze the intervention of a promotion of a healthy lifestyle program in overweight or obese former elite athletes that were inactive. Participants were divided into several sports, such as martial arts (25.6%), football (14.9%), athletics (mainly sprinters, middle- and long-distance track, and field) (14.9%), dancing/gymnastics (10.6%), swimming (8.5%), volleyball (9.6%), handball (5.3%), rugby (3.2%), and others (7.4%). For more details, the study protocol including recruitment and eligibility can be found elsewhere [26]. In total, 94 participants (63 males) (age: 43.1 ± 9.4; height 172.1 ± 8.9 cm; body mass 93.1 ± 17.9 kg; body mass index 31.1 ± 4.6 kg/m^2^) were included in the study. The study was approved by the Ethics Committee of the Faculty of Human Kinetics, University of Lisbon (Lisbon, Portugal) (CEFMH Approval Number: 16/2016) and was directed in accordance with the declaration of Helsinki for human studies from the World Medical Association. Additionally, the present study has been registered at www.clinicaltrials.gov (accessed on 3 June 2021) (clinicaltrials.gov ID: NCT03031951) prior to participants’ recruitment.

### 2.2. Procedures

Participants were weighted to the nearest 0.1 kg with a scale (Seca 876, Hamburg, Germany), and stature was measured to the closest 0.1 cm with a stadiometer (Seca, Hamburg, Germany). Body mass index was determined as body mass (kg) divided by height squared (m).

Bioelectrical resistance (R) and reactance (Xc) were obtained using a bioelectrical impedance spectroscopy (BIS) analyzer (model 4200B, Xitron Technologies, San Diego, CA, USA) at a frequency of 50 kHz. A detailed description is provided elsewhere [26].

DXA was performed in accordance with the standard practices recommended by the manufacturer on a Hologic Explorer-W, fan-beam densitometer (Hologic, Waltham, MA, USA) to obtain total whole-body fat mass, fat-free mass, and appendicular lean soft tissue mass, as described elsewhere [26]. Skeletal muscle mass was predicted according to Kim’s equation [9].

For upper-body strength, forearm maximal isometric strength was evaluated by a portable hand dynamometer JAMAR plus digital (Sammons Preston, Bolingbrook, IL, USA). Prior to the test, the grip dynamometer was adapted to the size of the hand of each subject. Then, the handgrip strength was assessed for the dominant hand. The assessment was performed with the subject standing up with the arms in a neutral position (halfway between supine and pronation position). Three attempts of 5 s of maximum contraction were allowed for the participants.

For lower-body strength, a horizontal leg-press isometric test (S0409, BPH) with the bent leg and the knee joint at an angle of 110° was completed. Participants performed 5 maximal voluntary repetitions lasting ~30 s. All participants were requested to generate strength the fastest they possibly could in all repetitions. The Plux software (Biosignalsplux) was utilized to evaluate the highest value among the maximal voluntary repetitions. The Valsalva maneuver was advised against during the test.

Maximal cardiorespiratory power was measured using the Bruce protocol test [27] performed on a variable speed and inclined treadmill (Quinton Treadmill, Model 640, 90TM Series). At the same time, the ventilated gas volumes, flow rates, and respiratory gas exchange were verified and pointed out by an open-circuit spirometry system (MedGraphics Corporation, Breezeex Software). When the respiratory exchange ratio was equal or greater than 1.10 for females and males aged 20–49 years old and equal or greater than 1.05 for females and males aged 50–64 years old and maximal oxygen uptake (VO2max) did not increase despite further grade increases, VO2max was achieved [28].

### 2.3. Statistical Analysis

Data analysis was performed using GraphPad Prism software v. 10 (GraphPad 189 Software, San Diego, California), SPSS v. 27.0 (SPSS, IBM Corp., Armonk, NY, USA), and G*Power v. 3.1.9.2 (G*Power, Stuttgart, Germany). For all tests, statistical significance was set at *p* < 0.05. The current sample size and power calculations were calculated for the Champ4Life trial to detect a moderate effect of changes in total body fat assessed by DXA, as described elsewhere [26]. However, the sample size for this cross-sectional study was assessed a posteriori considering a large effect size (appropriate for calculating effect size within a multiple regression model with continuous independent and dependent variables), with a 5% type I error and 80% power, which resulted in the appropriate sample size for conducting this cross-sectional study. Descriptive statistics (mean ± standard deviation) were calculated for all measurements. All variables were tested for normality using the Kolmogorov–Smirnov test. The Student’s t-test was used for independent samples to determine whether or not there were differences in PhA between male and female participants. Bivariate correlations were performed in preliminary analysis and the strength of the correlation coefficient (r) was evaluated, as suggested by Hopkins [29]; an r between 0 to 0.3 or 0 to −0.3, was considered small; 0.31 to 0.49 or −0.31 to −0.49, moderate; 0.5 to 0.69 or −0.5 to −0.69, large; 0.7 to 0.89 or −0.7 to −0.89, very large; and 0.9 to 1 or −0.9 to −1, near perfect for prediction of the relationship. Multiple regression analysis was used to determine if PhA was a significant predictor of skeletal muscle muscular strength and VO2max after correcting for confounding variables (age, sex, and fat mass).

## 3. Results

The general characteristics of the participants are shown in Table 1.

Baseline differences between males and females were found for all variables except for age and BMI.

**Figure 1:**
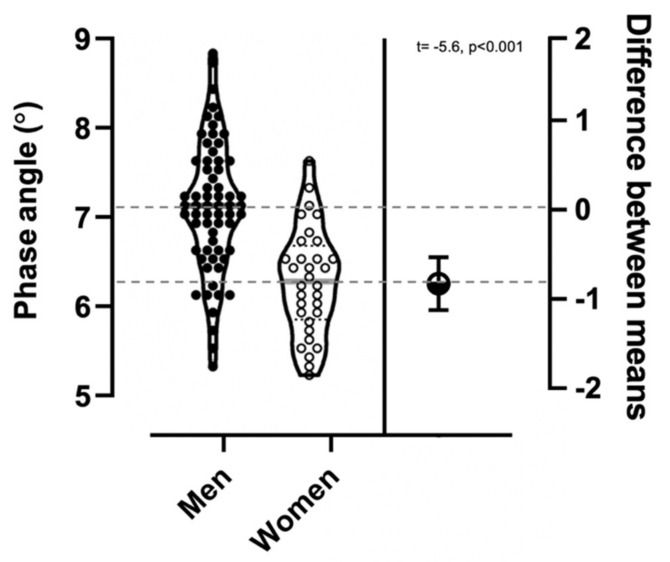
Violin plots showing the phase angle distribution in male and female participants; the result of the independent t-test is shown.

Significant differences (mean difference = −0.83, 95% CI = from −1.12 to −0.53, t = −5.61, *p* < 0.001) were measured for PhA between male and female participants, as shown in Figure 1.

**Figure 2:**
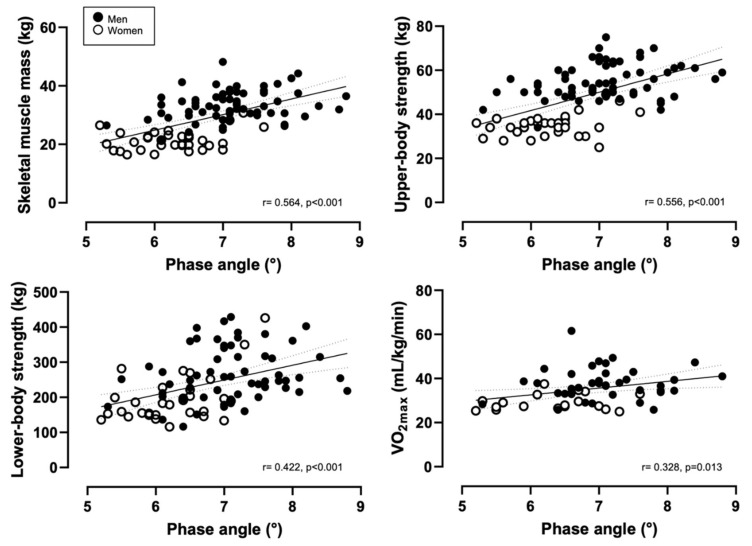
Scatterplots for the correlations between phase angle and skeletal muscle mass, upper- and lower-body strength, and maximum rate of oxygen consumption (V0_2max_).

There was a significant, near-perfect correlation between PhA and skeletal muscle mass, while a large correlation was found for PhA and upper-body strength, as shown in Figure 2. A moderate and positive correlation between PhA and lower-body and between PhA and VO_2max_ was found (Figure 2).

A multiple regression analysis was performed adjusting the relationship between PhA and skeletal muscle mass, upper- and lower-body strength, and VO2max for confounding variables (Table 2). PhA alone explained 32%, 31%, 12%, and 11% of skeletal muscle mass, upper- and lower body strength, and V02max variance, respectively. After adjusting for age and sex, PhA remained a significant predictor (Table 2). After adjusting for age, sex, and fat mass, PhA remained a significant predictor for skeletal muscle mass, upper- and lower-body strength but not for VO2max (Table 2).

## 4. Discussion

The present study was conducted to evaluate if PhA could be considered as a predictor of muscle quantity and strength and aerobic power in overweight/obese former athletes. The current outcomes showed that PhA accounted for more than 30% of skeletal muscle mass, remaining a significant predictor regardless of age sex, and fat mass. Indeed, fat mass was considered as a confounding variable since previous studies observed detrimental effects of excessive body fat to a loss of muscle mass and strength [21], as well as to a state of inflammation and therefore to a reduction in PhA [20]. In addition, PhA remained a borderline predictor of upper- and lower-body strength variance independently of age, sex, and fat mass. In contrast, although PhA alone was found to explain a small part of the VO2max variability, it lost its predictive power when adjusted for age, sex, and fat mass, likely given the smaller sample with maximal aerobic power assessed.

The participants of this study showed a PhA similar to the healthy general population with a median age of 43 years, for which the 50th percentile is identified as 6.9° and 6.3° for men and women, respectively [12]. Although the participants of this study were overweight or obese, the effects of sports practice in their lifespan may have allowed them to maintain a moderate amount of muscle mass and strength and therefore preserving PhA. However, a better interpretation of these values was not possible due to the absence of previous data on PhA in former athletes where higher values may be expected. Notably, obesity is a widespread problem among former athletes, especially for those who were engaged in speed-strength sport disciplines and weight-category sports, in comparison to aerobic endurance sports [30,31].

Preceding studies have linked PhA with sports performance and competitive level in athletes [15,16]. Recently, the relationship between PhA and sprint performance was shown, also highlighting how it is inversely correlated with perceived fatigue in soccer players [16]. Furthermore, in healthy athletes practicing similar disciplines, higher PhA values have been identified in players belonging to elite competitive levels [15,32,33]. As far as we know, although previous investigations aimed to study the role of PhA as a predictor of muscle strength in people affected by disease or elderly [13,34], our study was the first to relate PhA and muscle quantity and strength in former athletes. In this regard, it has been shown that PhA is directly related to the volume of intracellular fluids, which are expected to increase in close relationship with muscle tissue. For this reason, a higher PhA may be found in individuals with high muscle mass. Additionally, even if the direct association between mass and strength is currently debated, muscle mass is still one of the factors that contribute to the production of strength [2]. Regarding aerobic power, although an inverse relationship between body fat and VO2max was previously reported [18], PhA was not found to be a predictor of VO2max, after adjusting for sex, age, and fat mass, likely due to reduced number of individuals (n = 57) with VO2max assessed. Probably, cardiorespiratory factors turn out to be more decisive for aerobic power than the fluids distribution and the amount of muscle mass reflected by PhA [34,35].

The importance of muscle quantity and strength for health status is well established [2,36,37]. Muscle mass and strength have been shown to decrease with age, and this decline is mediated by body fat since this component reduces the contractile performance of skeletal muscle [2,21]. Not only the relationship between adiposity and metabolic disease is recognized but also the pathogenic potential of adipose tissue, particularly by excessive adipocyte hypertrophy, interacts with other body organs such as liver and skeletal muscle [38]. Thus, a better understanding of simple markers of skeletal muscle mass and strength in the current sample of former elite athletes with overweight and obesity is relevant since previous studies underlined obesity-related consequences in this population [39,40,41,42]. Since PhA is a direct derivative of the raw bioelectrical impedance parameters and given its association with mass and strength, it can be additionally considered as a predictor of skeletal muscle mass and strength, notwithstanding the careful attention that is required in data interpretation. Moreover, PhA reflects cellular integrity, and it facilitates the identification of conditions related to inflammation and accumulation of extracellular fluids, a scenario associated with obesity, which worsens in people exposed to an increased time of sedentary behavior [20,43,44].

It is important to address some limitations in this study. First, our results are generalizable to overweight and obese adults exposed to high levels of exercise in the past. Second, our results cannot be compared with those obtained from bioelectrical impedance measurements performed with different technologies and sampling frequencies other than 50 kHz. Finally, given the study’s cross-sectional nature, a cause–effect relationship could not be established, and the hypothesis generated by this study needs to be tested in experimental design studies.

## 5. Conclusions

The findings of this study highlight PhA as a possible predictor of skeletal muscle mass and isometric strength, regardless of age, sex, and body composition, suggesting its usefulness as a biomarker of muscle quantity and functionality in overweight/obese former athletes. PhA represents a novel and user-friendly bioelectrical impedance parameter for assessing body composition and strength in former athletes, but despite its prognostic relevance, it is still necessary to define valid cutoffs to use this marker as a clinical indicator.

## Figures and Tables

**Table 1:** Participants’ characteristics are shown as mean ± standard deviation.

Variable	Men (n = 62)	Women (n = 32)	All (n = 94)
Age (years)	42.8 ± 9.8	43.5 ± 8.7	43.1 ± 9.4
Body mass (kg) *	98.2 ± 17.9	81.7 ± 12.2	92.7 ± 18.0
Height (cm) *	175.9 ± 6.7	163.0 ± 6.3	171.6 ± 9.0
Body mass index (kg/m^2^)	31.7 ± 5.1	30.7 ± 3.9	31.4 ± 4.8
Fat mass (kg) *	27.6 ± 7.5	33.7 ± 6.9	33.7 ± 6.9
Fat mass (%) *	29.3 ± 5.5	43.0 ± 3.9	33.9 ± 8.2
Fat free mass (kg) *	70.6 ± 8.9	48.0 ± 3.9	60.3 ± 12.6
ALST (kg) *	28.9 ± 4.5	18.8 ± 2.9	25.4 ± 6.3
Skeletal muscle mass (kg) *	33.4 ± 5.2	21.3 ± 3.4	29.3 ± 7.4
Resistance (ohm) *	439.6 ± 47.3	561.6 ± 69.7	480.2 ± 80.1
Reactance (ohm) *	54.5 ± 7.7	61.2 ± 7.4	56.7 ± 8.2
Phase angle (°)*	7.1 ± 0.7	6.2 ± 0.6	6.8 ± 0.8
Upper-body strength (kg) *	55.1 ± 8.0	35.2 ± 5.1	48.4 ± 11.9
Lower-body strength (kg) *	264.5 ± 74.7	196.1 ± 67.7	241.4 ± 79.4
VO_2max_ (mL/kg/min) *^,#^	38.0 ± 7.2	29.3 ± 3.7	35.2 ± 7.4

Abbreviations: ALST, appendicular lean soft tissue; VO_2max_, maximum rate of oxygen consumption; * differences between gender. ^#^ VO_2max_ valid data was obtained in 57 participants (total).

**Table 2:** Unadjusted and adjusted models using phase angle as the independent variable for determining skeletal muscle mass, upper- and lower-body strength, and VO_2max_.

Model	R^2^	Std. Error	β	95% CI	*p*-value
Skeletal muscle mass					
Phase angle	0.318	6.11	5.338	3.71, 6.95	<0.001
Model 1 ^a^	0.664	4.33	1.410	−0.01, 2.82	0.050
Model 1 ^b^	0.752	3.74	2.158	0.91, 3.40	0.001
Upper-body strength					
Phase angle	0.309	9.90	8.315	5.75, 10.87	<0.001
Model 1 ^a^	0.701	6.59	2.245	0.44, 4,47	0.018
Model 1 ^b^	0.708	6.60	2.846	0.64, 5.05	0.012
Lower-body strength					
Phase angle	0.115	79.73	35.782	15.09, 56.47	0.001
Model 1 ^a^	0.275	68.75	22.935	1.32, 44.54	0.038
Model 1 ^b^	0.275	69.509	24.209	1.02, 47.39	0.041
VO_2max_					
Phase angle	0.107	7.11	3.090	0.682, 5.49	0.013
Model 1 ^a^	0.310	6.33	0.757	−1.79, 3.31	0.555
Model 1 ^b^	0.655	4.52	−0.163	−3.03, 0.47	0.098

Abbreviations: R^2^, coefficient of determination; β, unstandardized coefficients beta; CI, confidence interval; Std. Error, standard error; VO_2max_, maximum rate of oxygen consumption. ^a^, adjusted for age and sex; ^b^, adjusted for age, sex, and fat mass.

## Data Availability

The data that support the findings of this study are available from the corresponding author upon reasonable request.
